# Appendicitis inflammatory response versus pediatric appendicitis score for grading disease severity in children

**DOI:** 10.1038/s41598-026-42122-w

**Published:** 2026-04-01

**Authors:** Husam Ibrahimoglu

**Affiliations:** Department of Pediatric Surgery, Kütahya City Hospital, Kütahya Health Sciences University, Kütahya, Turkey

**Keywords:** Imaging findings, Complicated appendicitis, Pediatric appendicitis, Appendicitis inflammatory response score, Pediatric appendicitis score, Diseases, Gastroenterology, Health care, Medical research, Risk factors

## Abstract

Acute appendicitis is a frequent surgical emergency in children, and early recognition of severe forms remains challenging. This prospective observational study compared the Appendicitis Inflammatory Response (AIR) and Pediatric Appendicitis Score (PAS) for grading disease severity in pediatric acute appendicitis. Among 542 children assessed, 138 with suspected appendicitis were included, and 136 underwent appendectomy. All included patients were prospectively scored with AIR and PAS, and clinical, radiologic, intraoperative, and histopathologic findings were recorded. Associations between score categories and complicated appendicitis (gangrenous, abscess, or diffuse peritonitis) or perforated appendicitis were analyzed, and diagnostic performance was assessed using receiver operating characteristic curves. Higher AIR and PAS categories were associated with increasing appendix diameter; AIR categories were significantly associated with both complicated appendicitis and perforation, whereas PAS categories were significantly associated only with perforation. For complicated appendicitis, the area under the curve (AUC) was slightly higher for AIR than PAS, whereas for perforation both scores showed similar AUCs. These findings suggest that AIR is more informative than PAS for overall grading of disease severity in pediatric acute appendicitis, while AIR and PAS provide comparable, moderate accuracy for predicting perforation.

## Introduction

 Acute appendicitis (AA) is one of the most common surgical emergencies in children. One study reported an incidence of AA of 228 per 100,000 cases^1^. Complicated appendicitis (CA) accounts for approximately 30% of all appendicitis cases. CA is defined intraoperatively as the presence of a visible appendiceal perforation, gangrenous tissue, fibrinopurulent fluid, intra-abdominal abscess, or extraluminal fecalith^2,3^. While perforation is most often identified based on intraoperative findings, some studies have instead defined appendiceal perforation according to histopathological criteria^4^. However, one study emphasized that it is more appropriate to identify appendiceal perforation based on intraoperative findings^5^.

For many years, the diagnosis of AA relied solely on history, physical examination, and laboratory parameters such as white blood cell count (WBC), neutrophil percentage (NEU%), and C-reactive protein (CRP); this may have contributed to increased perforation rates. With the introduction of ultrasonography (USG) and computed tomography (CT) in the diagnosis of AA, the negative appendectomy rate has decreased, but the perforation rate remains high^6^. However, the use of CT in the diagnosis of AA in children is known to carry a risk of radiation exposure and to increase healthcare costs^7,8^.

Various scoring systems have been developed for the diagnosis of AA to reduce the number of negative appendectomies in children, limit the use of CT scans, and conserve logistical and financial resources^9^. The Pediatric Appendicitis Score (PAS), comprising 10 variables, was defined by Samuel in 2002 to predict AA. The PAS score is largely based on history and physical examination findings, and because it does not include inflammatory parameters such as CRP, its role in predicting the severity of AA and its complicated course is not fully clear^10^. In contrast, the Appendicitis Inflammatory Response (AIR) score was developed by Andersson in 2008 to identify CA. It includes both clinical findings and inflammatory markers such as CRP, is based on 12 parameters, and classifies patients into three risk groups (low, intermediate, and high)^11^.

The number of studies in the literature that have simultaneously compared AIR and PAS scores with radiological, intraoperative, and histopathological findings—particularly for CA and PA in childhood—is limited.

This study evaluated the relationship between AIR and PAS scores and radiological, intraoperative, and histopathological findings in predicting CA and PA. The aim was to determine which score provides the strongest prediction of disease severity to support clinical decision-making.

## Materials and methods

### Study design and setting

This prospective observational study was conducted in the Pediatric Emergency Department of a tertiary university hospital between April 2023 and February 2024. The aim of the study was to compare the performance of the Pediatric Appendicitis Score (PAS) and Appendicitis Inflammatory Response (AIR) scores in determining the severity of acute appendicitis (AA), including complicated and perforated forms, and to evaluate their relationship with radiologically measured appendix diameter. The primary endpoint was intraoperative complicated appendicitis, whereas histopathologic perforated appendicitis was evaluated as a secondary endpoint.

### Patient selection

A total of 542 children aged 0–18 years who presented with abdominal pain during the study period were evaluated. Of these, 138 children with suspected acute appendicitis after the initial evaluation were included in the study. Patients with diagnoses other than acute appendicitis or with incomplete clinical and/or laboratory data were excluded.

### Data collection

#### Demographic and clinical findings

Demographic characteristics, including age and gender, as well as the duration of symptoms before presentation, were recorded in detail for all patients. Physical examination findings were systematically documented using a standardized form.

#### Laboratory examinations

White blood cell count (WBC, ×10³/µL), neutrophil percentage (NEU%), and C-reactive protein (CRP, mg/L) were measured from venous blood samples obtained on admission.

#### Imaging modalities

Imaging was performed according to clinical need. Ultrasonography (USG) was the first-line imaging modality at our center, and computed tomography (CT) was used in cases where USG was inconclusive or when complications were suspected. Approximately 20% of the patients were referred from external centers. In some of these patients, CT imaging had already been performed externally and was not repeated at our center. An appendix diameter of 6 mm or greater was considered consistent with acute appendicitis on imaging evaluation^6^.

### Scoring systems

#### AIR score

Patients were divided into three risk groups based on their AIR score: Group A, low risk (0–4); Group B, intermediate risk (5–8); and Group C, high risk (9–12) (Table 1).

**Table 1:** Appendicitis inflammatory response (AIR) score^11^.

Diagnostic parameter	Score
Nausea or vomiting	1
Right lower quadrant pain	1
Mild tenderness, guarding, and rebound Moderate tenderness, guarding, and rebound Severe tenderness, guarding, and rebound	1 2 3
Temperature ≥ 38.5 °C	1
Polymorphonuclear leukocytes 70–84% Polymorphonuclear leukocytes ≥ 85%	1 2
WBC count 10.0–14.9 × 10⁹/L WBC count ≥ 15 × 10⁹/L	1 2
CRP 10–49 mg/L CRP ≥ 50 mg/L	1 2
Interpretation 0–4, low probability: outpatient follow-up if no alarming signs 5–8, moderate probability: hospital observation, imaging, and laparoscopy 9–12, high probability: surgical intervention	

#### PAS score

The PAS score was calculated on a scale from 0 to 10. For the purposes of this study, patients were classified into three risk groups to align with the statistical analysis: Group A: Low risk (0–4), Group B: Intermediate risk (5–7) and Group C: High risk (8–10) (Table 2).

**Table 2:** Pediatric appendicitis score (PAS)^10^

Diagnostic parameter	Score
Tenderness increased by coughing or hopping	2
Anorexia	1
Fever	1
Nausea or vomiting	1
Right lower quadrant tenderness	2
Leukocytosis	1
Neutrophilia	1
Migration of pain	1
Interpretation: PAS score ≥ 6 implies a high likelihood of acute appendicitis.	

### Surgical decision and histopathological evaluation

All surgical decisions were made by a single pediatric surgeon with approximately 10 years of experience in pediatric surgery. The macroscopic appearance of the appendix (simple vs. complicated) was recorded in a standardized manner during surgery.

### Intraoperative definition of simple and complicated appendicitis

In accordance with international pediatric surgical guidelines, Intraoperatively, simple appendicitis was defined as an inflamed but non-gangrenous appendix without visible perforation, periappendicular abscess, fecalith spillage, or free pus in the peritoneal cavity; the presence of a small amount of clear reactional fluid alone was not considered a sign of complicated disease. Complicated appendicitis was defined by the presence of at least one of the following intraoperative findings: perforation of the appendix; gangrenous appendicitis; obstruction by a fecalith with local peritonitis; periappendicular abscess or purulent collection; or free pus in the abdominal cavity^2,3^. 

### Histopathological definition of acute and perforated appendicitis

On histopathological examination, documentation of perforation in the appendiceal wall or disruption of wall integrity due to transmural necrosis was considered perforated appendicitis^5^. Cases without perforation but with histopathological findings consistent with acute inflammation were classified as acute (non-perforated) appendicitis.

### Ethical approval

The study was approved by the Kütahya Health Sciences University Clinical Research Ethics Committee (Decision No: 2023-06/03, Date: April 11, 2023). Written informed consent was obtained from the parents of all patients. All methods were performed in accordance with the relevant guidelines and regulations and with the principles of the Declaration of Helsinki.

### Study flow

**Fig. 1:**
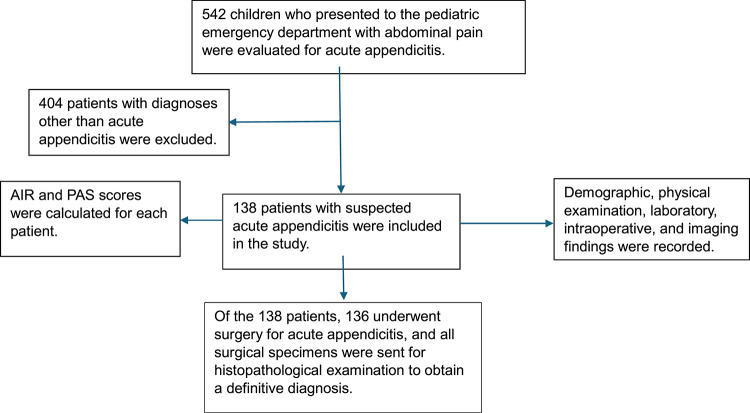
Flow diagram of patient selection and diagnostic evaluation in children with suspected acute appendicitis.

A schematic representation of the patient selection process, scoring assessments, and diagnostic confirmation is presented in Fig. 1.

### Statistical analysis

Distributional characteristics of continuous variables were assessed using the Shapiro–Wilk test. Continuous data are presented as mean ± standard deviation (minimum–maximum), and categorical variables are presented as numbers and percentages.

In comparing acute–perforated and simple–complicated appendicitis groups, Student’s t-test was used for normally distributed variables and the Mann–Whitney U test was used for non-normally distributed variables. Categorical variables were compared using the chi-square test or, where appropriate, Fisher’s exact test. WBC, NEU%, and CRP values were compared across AIR and PAS risk groups (A, B, C) using the Kruskal–Wallis test, and data were summarized as medians with interquartile ranges (IQRs). The relationship between the AIR and PAS score groups and radiological measurements (appendiceal diameter) was assessed using the Kruskal–Wallis test.

The predictive performance of the AIR and PAS scores for complicated and perforated appendicitis was evaluated using ROC analysis. Area under the curve (AUC), 95% confidence intervals, and optimal cutoff points were calculated using the Youden index. Comparisons of independent ROC curves were performed using MedCalc Statistical Software (MedCalc Software Ltd., Version 23.0.9). ROC *p* values ​​represent the comparison of the AUC with the null hypothesis (AUC = 0.5).

The agreement of AIR and PAS scores was analyzed using the kappa coefficient (κ). Kappa coefficients < 0.20 were interpreted as slight agreement, 0.21–0.40 as fair agreement, 0.41–0.60 as moderate agreement, 0.61–0.80 as good agreement, and > 0.80 as excellent agreement.

SPSS for Windows, Version 24.0 (IBM Corp., Armonk, NY, USA) was used for all other analyses. Statistical significance was set at *p* < 0.05.

## Results

In this study, 138 pediatric patients evaluated with a preliminary diagnosis of acute appendicitis were analyzed. Of the patients, 67.6% were male, and the mean age was 11.22 ± 3.36 years. The mean duration of symptoms before presentation was 1.52 ± 1.03 days, and the mean length of hospital stay was 3.60 ± 1.79 days. Regarding imaging modalities, CT was used in 44.9% of patients, USG in 32.6%, and both modalities in 22.5%. The mean appendix diameter was 7.75 ± 3.01 mm. On laboratory evaluation, the mean WBC was 14.26 ± 5.29 × 10³/µL, mean NEU% was 76.78 ± 14.01, and mean CRP was 35.84 ± 43.93 mg/L. The mean AIR and PAS scores were 7.28 ± 2.14 and 6.91 ± 1.68, respectively (Table 3).

**Table 3:** Demographic, laboratory, and radiologic findings.

Variable	*n* (%)	Mean ± SD (Min–Max)
Male	94 (67.6%)	
Female	44 (31.7%)	
Age (years)		11.22 ± 3.36 (4.00–18.00)
Symptom duration (days)		1.52 ± 1.03 (1.00–8.00)
Length of stay (days)		3.60 ± 1.79 (2.00–17.00)
Imaging modality and Appendix diameter		
CT	62 (44.9%)	
USG	45 (32.6%)	
USG + CT	31 (22.5%)	
Appendix diameter (mm)		7.75 ± 3.01
Laboratory findings		
WBC (×10³/µL)		14.26 ± 5.29
CRP (mg/L)		35.84 ± 43.93
NEU (%)		76.78 ± 14.01
Scores		
AIR score		7.28 ± 2.14
PAS score		6.91 ± 1.68

SD, standard deviation. *N* = 138.

Of the 138 patients, 136 underwent appendectomy; two patients were discharged without surgery and did not re-present with acute appendicitis during follow-up. A total of three postoperative complications were observed: two patients developed intra-abdominal abscess, which resolved with conservative treatment; one patient developed postoperative intussusception, which regressed spontaneously within five days without the need for any additional intervention.

On histopathological examination, acute appendicitis was detected in 79.4% (*n* = 108) and perforated appendicitis in 16.2% (*n* = 22) of cases. Lymphoid hyperplasia (*n* = 4), chronic appendicitis (*n* = 1), and negative appendectomy (*n* = 1) were observed at lower frequencies. Intraoperatively, 72.1% (*n* = 98) of patients were classified as having simple appendicitis and 27.9% (*n* = 38) as complicated appendicitis (Table 4).

**Table 4:** Intraoperative and histopathological findings.

Variable	*n* (%)
Histopathological diagnosis	
Acute appendicitis	108 (79.4)
Perforated appendicitis	22 (16.2)
Lymphoid hyperplasia	4 (2.9)
Chronic appendicitis	1 (0.7)
Negative appendectomy	1 (0.7)
Intraoperative diagnosis	
Simple appendicitis	98 (72.1)
Complicated appendicitis	38 (27.9)

*N* = 136.

When laboratory and radiological variables were compared between patients with complicated and simple appendicitis, only NEU% was significantly higher in the CA group (*p* = 0.032). Although the appendix diameter was greater in the complicated group, this difference was not statistically significant (*p* = 0.925). In the perforation analysis, however, appendix diameter was significantly higher in perforated cases (*p* = 0.020) (Table 5).

**Table 5:** Relationship between laboratory/radiologic findings and CA / PA.

Variable	CA (mean)	Simple AA (mean)	*p* value (CA)	PA (mean)	Non-perforated AA (mean)	*p* value (PA)
WBC ×10³/µL	15.7	13.72	0.074	15.67	14.0	0.191
NEU %	80.56	75.36	0.032**	81.7	75.86	0.226
CRP mg/L	41.47	33.71	0.213	31.41	36.67	0.591
Appendix diameter (mm)	8.78	8.74	0.925	9.42	8.56	0.020**

**p values were obtained using the Mann–Whitney U test; *p* < 0.05 was considered statistically significant. AA, acute appendicitis; CA, complicated appendicitis; PA, perforated appendicitis.

When laboratory parameters were compared across AIR risk categories, there was a clear stepwise increase in WBC, NEU% and CRP from AIR group A to C. Median WBC values were 7.37 (IQR 6.03–11.91), 12.76 (9.77–17.35) and 16.88 (14.31–19.88) ×10³/µL in AIR groups A, B and C, respectively (Kruskal–Wallis *p* = 0.00001). Median NEU% increased from 61.15 (45.88–75.47) to 79.00 (69.80–84.20) and 83.85 (80.00–87.97) (*p* < 0.001), and median CRP from 4.50 (2.43–7.88) mg/L to 13.00 (3.10–42.30) mg/L and 29.00 (11.62–60.35) mg/L (*p* = 0.003). For PAS, median WBC and NEU% also increased across score groups (*p* = 0.012 and *p* = 0.005, respectively), whereas differences in CRP between PAS groups were not statistically significant (*p* = 0.16). (Tables 6 and 7)

**Table 6:** Inflammatory markers according to AIR score groups.

AIR group	WBC ×10³/µL Median (IQR)	NEU % Median (IQR)	CRP mg/L Median (IQR)
A	7.37 (6.03–11.91)	61.15 (45.88–75.47)	4.50 (2.43–7.88)
B	12.76 (9.77–17.35)	79.00 (69.80–84.20)	13.00 (3.10–42.30)
C	16.88 (14.31–19.88)	83.85 (80.00–87.97)	29.00 (11.62–60.35)
*p* value	0.00001^**^	< 0.001^**^	0.003^**^

**p values were calculated using the Kruskal–Wallis test. *p* < 0.05 was considered statistically significant. WBC, white blood cell count; NEU%, neutrophil percentage; CRP, C-reactive protein.

**Table 7:** Inflammatory markers according to PAS score groups.

PAS group	WBC ×10³/µL Median (IQR)	NEU % Median (IQR)	CRP mg/L Median (IQR)
A	8.38 (6.45–11.83)	63.10 (47.00–68.00)	4.50 (2.50–14.00)
B	14.11 (10.44–18.48)	82.20 (73.00–87.80)	21.10 (4.90–42.00)
C	14.95 (12.16–19.58)	80.80 (75.00–85.40)	18.10 (3.50–67.50)
*p* value	0.012	0.005^**^	0.16

**p values were calculated using the Kruskal–Wallis test. *p* < 0.05 was considered statistically significant. WBC, white blood cell count; NEU%, neutrophil percentage; CRP, C-reactive protein.

Radiologically, appendix diameter could not be measured in 30 patients. In one patient, perforated Meckel’s diverticulum was reported in the external CT report, whereas complicated appendicitis was found intraoperatively. Among the 107 patients in whom appendix diameter could be measured, appendix diameter showed a stepwise increase across both AIR and PAS risk groups. According to AIR score, the mean appendix diameter was 7.57 ± 1.72 mm in Group A (median 7.0; Q1–Q3: 6.5–8.0), 8.20 ± 1.99 mm in Group B (median 8.0; Q1–Q3: 7.0–9.0), and 9.72 ± 3.55 mm in Group C (median 9.0; Q1–Q3: 7.0–11.25). This upward trend across AIR groups was at the borderline of significance but did not reach statistical significance (*p* = 0.109). Similarly, according to PAS score, the mean appendix diameter was 7.00 ± 0.89 mm in PAS Group A (median 7.0; Q1–Q3: 6.25–7.75), 8.26 ± 2.30 mm in Group B (median 8.0; Q1–Q3: 7.0–10.0), and 9.88 ± 3.36 mm in Group C (median 9.0; Q1–Q3: 8.0–11.0). The difference in appendix diameter across PAS groups was statistically significant (*p* = 0.015) (Table 8).

**Table 8:** Appendix diameter according to AIR and PAS score groups.

Score system	Group	*n*	Mean ± SD (mm)	Median (Q1–Q3), mm	*p* value
AIR	A	7	7.57 ± 1.72	7.0 (6.5–8.0)	0.109
AIR	B	41	8.20 ± 1.99	8.0 (7.0–9.0)	
AIR	C	32	9.72 ± 3.55	9.0 (7.0–11.25)	
PAS	A	6	7.00 ± 0.89	7.0 (6.25–7.75)	0.015**
PAS	B	45	8.26 ± 2.30	8.0 (7.0–10.0)	
PAS	C	29	9.88 ± 3.36	9.0 (8.0–11.0)	

**p values were calculated using the Kruskal–Wallis test. *p* < 0.05 was considered statistically significant.

When the relationship between AIR and PAS score groups and acute versus perforated appendicitis was examined, the distribution of AIR scores differed significantly between the acute and perforated groups (*p* = 0.029), whereas no statistically significant difference was observed between PAS groups (*p* = 0.1165). In the comparison of simple versus complicated appendicitis, AIR scores differed significantly between groups (*p* = 0.007), while PAS scores did not show a significant difference (*p* = 0.2137) (Tables 9 and 10).

**Table 9:** Comparison of AIR and PAS score groups with acute and perforated appendicitis.

AIR group	Acute (n)	Perforated (n)	*p* value
AIR-A	11	1	0.029**
AIR-B	65	8	
AIR-C	32	13	

PAS group	Acute (n)	Perforated (n)	*p* value
PAS-A	9	0	0.1165
PAS-B	62	10	
PAS-C	37	12	

**p values were calculated using the chi-square test. *p* < 0.05 was considered statistically significant.

**Table 10:** Comparison of AIR and PAS score groups with simple and complicated appendicitis.

AIR group	Simple (n)	Complicated (n)	*p* value
AIR-A	11	3	0.007**
AIR-B	61	14	
AIR-C	26	21	

PAS group	Simple (n)	Complicated (n)	*p* value
PAS-A	9	2	0.2137
PAS-B	58	18	
PAS-C	31	18	

**p values were calculated using the chi-square test. *p* < 0.05 was considered statistically significant.

**Fig. 2:**
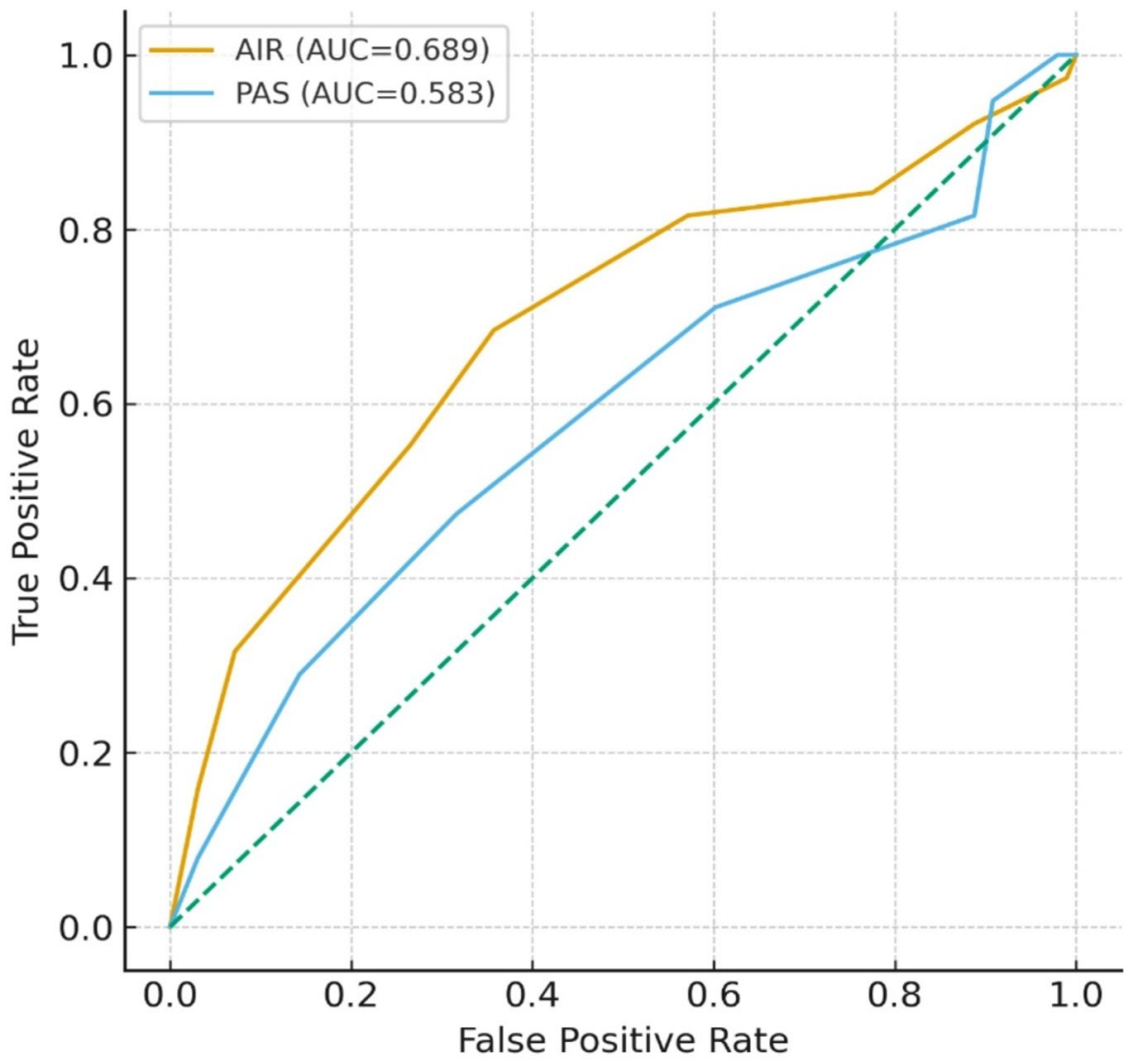
ROC curves of AIR and PAS scores for predicting CA in children. AUC and other diagnostic performance measures are presented in Table 11.

In ROC analysis for prediction of complicated appendicitis, the AUC of the AIR score was 0.641 (95% CI 0.538–0.743), whereas the AUC of the PAS score was 0.622 (95% CI 0.518–0.725) (Table 11; Fig. 2).

**Table 11:** ROC analysis for complicated appendicitis.

Score	Cut-off	AUC	95% CI	Sensitivity (%)	Specificity (%)	PPV (%)	NPV (%)	Youden
AIR	> 8	0.641	0.538–0.743	60.8	67.7	41.6	82.2	0.285
PAS	> 6.5	0.622	0.518–0.725	79.4	42.7	35.5	84.0	0.221

AUC, area under the curve; CI, confidence interval; PPV, positive predictive value; NPV, negative predictive value. ROC analyses were performed using the Youden index. AUC values are presented with 95% confidence intervals.

**Fig. 3:**
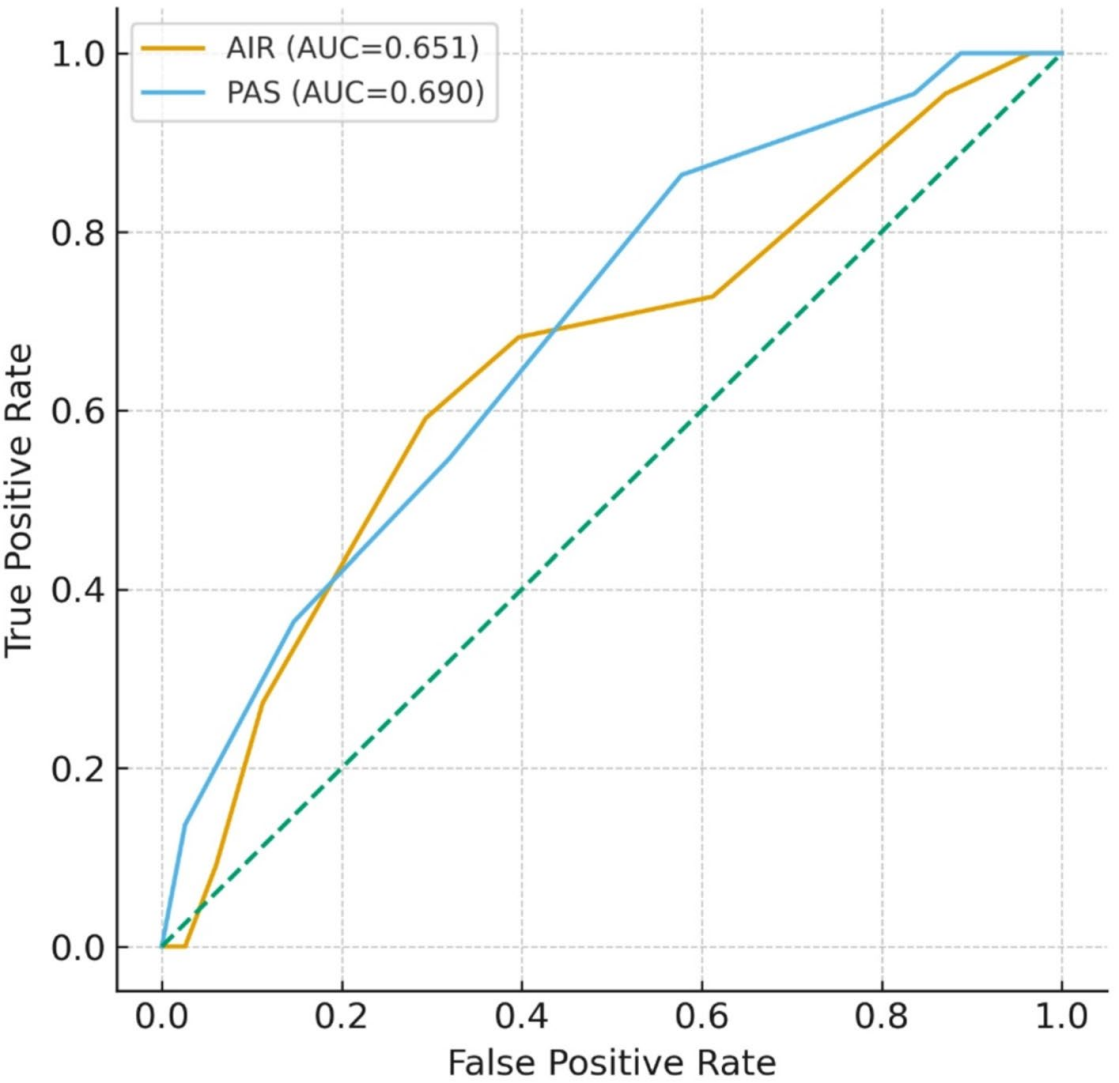
ROC curves of AIR and PAS scores for predicting PA in children. AUC and other diagnostic performance measures are presented in Table 12.

For prediction of perforated appendicitis, the AUC was 0.642 (95% CI 0.509–0.774) for AIR and 0.673 (95% CI 0.552–0.793) for PAS. The optimal cut-off values were > 8 for AIR and > 6.5 for PAS (Table 12; Fig. 3).

**Table 12:** ROC analysis for perforated appendicitis.

Score	Cut-off	AUC	95% CI	Sensitivity (%)	Specificity (%)	PPV (%)	NPV (%)	Youden
AIR	> 8	0.642	0.509–0.774	59.1	70.4	28.9	89.4	0.295
PAS	> 6.5	0.673	0.552–0.793	86.4	38.9	22.4	93.3	0.253

AUC, area under the curve; CI, confidence interval; PPV, positive predictive value; NPV, negative predictive value. ROC analyses were performed using the Youden index. AUC values are presented with 95% confidence intervals.

 In kappa analysis evaluating the agreement of AIR and PAS scores in predicting complicated and perforated appendicitis, the kappa coefficient for complicated appendicitis was 0.243 (*p* = 0.0046) for AIR and 0.116 (*p* = 0.217) for PAS. For perforation, kappa was 0.206 (*p* = 0.0413) for AIR and 0.133 (*p* = 0.067) for PAS (Table 13).

**Table 13:** Kappa agreement analysis – agreement of AIR and PAS scores.

Outcome evaluated	AIR kappa (κ)	AIR *p* value	PAS kappa (κ)	PAS *p* value
Complicated appendicitis	0.243	0.0046**	0.116	0.217
Perforated appendicitis	0.206	0.0413**	0.133	0.067

κ: kappa statistic. Kappa analysis was performed using Cohen’s kappa method. ** *p* < 0.05 was considered statistically significant.

The agreement between the diagnoses of complicated and perforated appendicitis themselves was moderate, with a kappa value of 0.333 (Table 14).

**Table 14:** Kappa agreement analysis between complicated and perforated appendicitis diagnoses.

Complicated = 0	Perforated (1)	Non-perforated (0)	Kappa (κ)
	8	93	0.333
Complicated = 1	14	24	

κ: kappa statistic. Kappa analysis was performed using Cohen’s kappa method.

## Discussion

Acute appendicitis (AA) remains one of the most common surgical emergencies; its global incidence is approximately 228 per 100,000 individuals and is highest among males aged 15–19 years^1,12,13^. Inflammatory markers, such as WBC, NEU%, and CRP, are recognized predictors of CA^14^. In our study, WBC and CRP levels did not differ significantly between CA and simple appendicitis patients, whereas NEU% was significantly higher in CA patients, consistent with prior studies^15^.

In our study, when the relationship between AIR and PAS score groups and disease severity was evaluated, the AIR score—particularly the high-risk C group—was more effective in predicting both CA and PA. This is consistent with previous reports showing that AIR, which incorporates CRP and NEU% as graded inflammatory markers, provides better diagnostic performance for CA than purely clinical scores^11,15–17^. In our cohort, increasing AIR categories were associated with a progressive rise in WBC, NEU%, and CRP, supporting the close alignment between AIR and the underlying inflammatory response. This pattern may partly explain why AIR performs better than PAS in grading disease severity.

By contrast, PAS demonstrated more limited performance in predicting CA and PA. Although the probability of acute appendicitis is known to increase with a PAS score ≥ 6, its ability to discriminate complicated disease is less clear and often depends on a PAS score > 8 and additional information such as CRP and USG findings^10,18,19^. One important reason is that PAS relies mainly on clinical symptoms and treats leukocytosis and neutrophilia as simple dichotomous items, without explicitly incorporating CRP or other graded inflammatory values. As a result, even when WBC and NEU% are substantially elevated, PAS may only partially capture the true inflammatory burden and may underestimate disease severity in some children. Taken together, these data support the notion that AIR can be regarded as a refined, more advanced version of PAS, integrating clinical findings with graded inflammatory markers to more accurately mirror the underlying inflammatory burden and severity of acute appendicitis in children.

Although graded-compression USG is the first-line imaging modality for diagnosing AA, several studies have reported increased diagnostic accuracy with computed CT^20^. In our study, the relatively high frequency of CT use was largely attributable to the fact that approximately 20% of patients were referred from external centers, and many had already undergone CT prior to presentation. Appendix diameter, an important imaging parameter, has been shown to be greater in patients with CA or PA compared to those with uncomplicated disease^21^. In our cohort, appendix diameter was higher in the CA and PA groups compared to the simple and non-perforated groups; however, this difference reached statistical significance only in the PA group (*p* = 0.020) and not in the CA group. In addition, appendix diameter tended to be higher in the high-risk AIR group; however, this trend did not reach statistical significance (*p* = 0.109). Conversely, a significant association was observed between high-risk PAS scores and appendix diameter (*p* = 0.015). Taken together, these findings suggest that the AIR score may help identify CA and PA even in the absence of clear radiologic findings, whereas PAS performs better when supported by imaging. This interpretation aligns with previous studies indicating that AIR-based risk stratification may reduce the need for diagnostic imaging, whereas PAS should more often be used in conjunction with imaging to predict CA or PA^9,11,19^. Consistent with this, appendix diameter increased across AIR and PAS risk groups; however, this association reached statistical significance only for PAS. This pattern suggests that the diagnostic performance of PAS is more closely tied to pronounced radiologic enlargement of the appendix, whereas AIR can differentiate CA or PA cases from uncomplicated appendicitis even when appendix diameter is only modestly increased, supporting its use as a more robust indicator of early disease severity.

When the performance of the AIR score and of PAS in predicting CA and PA were compared, AIR performed better than PAS in predicting CA, whereas both scores demonstrated similar diagnostic capacity for PA. ROC analysis exhibited that, for CA, the area AUC was 0.641 for AIR and 0.622 for PAS, whereas for PA the AUC values were 0.642 for AIR and 0.673 for PAS. The slightly higher AUC of PAS compared to AIR in predicting PA did not translate into clinically meaningful superiority. This pattern is consistent with the findings of Porgorelić et al.^22^, who reported high sensitivity (89.5%) and good specificity (71.9%) for AIR using a cut-off value of > 9. Similarly, several studies have emphasized that AIR provides better diagnostic accuracy than the Alvarado score and PAS^23^. In our study, the relatively low AUC, sensitivity, and specificity values are likely related to the limited sample size, particularly the small number of CA and PA cases.

Although several studies suggest that PAS alone may be useful in assessing the severity of AA, numerous studies indicate that PAS is more effective in distinguishing CA only when evaluated together with CRP and USG findings^18,19,24,25^. Taken together, these findings suggest that AIR can detect CA before perforation develops, whereas PAS becomes informative predominantly in the presence of PA, indicating that PAS is less effective as a stand-alone tool for clinical decision-making about disease severity.

When we assessed the agreement between each scoring system and the diagnoses of CA and PA, AIR demonstrated only fair agreement, whereas PAS exhibited slight or non-significant agreement. This likely reflects the reliance of PAS on history and physical examination findings, which may be difficult to obtain accurately, particularly in younger children^19^. In contrast, AIR can be considered a more robust scoring system for CA and PA, as it incorporates inflammatory laboratory parameters (CRP and NEU%) in addition to clinical findings^16^. Notably, the agreement between CA and PA diagnoses themselves was only moderate (kappa = 0.333). Several authors have emphasized that CA or PA should be defined based primarily on intraoperative findings rather than on histopathological diagnosis^5^.

The prospective design of our study, along with the simultaneous evaluation of AIR and PAS scores in the same patient cohort and the integration of radiologic, clinical, and histopathological findings of CA and PA, represents a key strength. However, major limitations include the relatively small overall sample size—particularly the limited number of CA and PA patients—the single-center design, and the inclusion of patients referred from external centers who had already undergone CT imaging. Consequently, multicenter studies with larger cohorts are needed to validate and extend these findings.

As discussed above, an important limitation of our study is the use of two different reference standards for AIR and PAS—intraoperative findings for complicated appendicitis and histopathological perforation for perforated appendicitis. Because these outcomes only partially overlap, this may have contributed to discrepancies between the scores and may limit the generalizability of our findings. Therefore, multicenter studies with larger sample sizes are needed to confirm and expand upon our findings. Finally, we did not perform a separate ROC analysis focusing on low-score ranges (e.g., AIR 0–4) to formally evaluate the ‘rule-out’ performance of the scores; this question should be addressed in future studies with larger cohorts. In addition, long-term follow-up of the entire cohort of 542 children presenting with abdominal pain was not systematically performed, and therefore late diagnoses of appendicitis outside our predefined follow-up window cannot be completely excluded.

## Conclusion

Our findings demonstrate that the AIR score outperforms PAS in predicting CA, whereas both scores exhibit similar diagnostic capacity for PA. Although PAS-based assessments of CA or PA should generally be interpreted alongside radiologic imaging, a high AIR score can predict CA and PA even in the absence of clear radiologic findings. Overall,  the AIR score appears to be a more reliable tool than PAS for evaluating disease severity in pediatric AA and for identifying CA patients. These findings suggest that the AIR score may be useful as a first-line clinical risk stratification tool to guide decision-making and potentially reduce unnecessary imaging in children with suspected acute appendicitis.

## Data Availability

The datasets generated and/or analysed during the current study are not publicly available due to the Personal Data Protection Law of the Republic of Turkey, but are available from the corresponding author on reasonable request.
